# Comparing Characteristics of Patients Who Connect Their iPhones to an Electronic Health Records System Versus Patients Who Connect Without Personal Devices: Cohort Study

**DOI:** 10.2196/14871

**Published:** 2019-08-22

**Authors:** William J Gordon, David W Bates, Daniel Fuchs, John Pappas, Sara Silacci, Adam Landman

**Affiliations:** 1 Department of Medicine, Brigham and Women's Hospital Boston, MA United States; 2 Harvard Medical School Boston, MA United States; 3 Partners HealthCare Somerville, MA United States; 4 Massachusetts General Hospital Boston, MA United States; 5 Department of Emergency Medicine, Brigham and Women's Hospital Boston, MA United States

**Keywords:** health information interoperability, patient participation, information technology, mobile health

## Abstract

**Background:**

While individual access to health records has traditionally been through paper and other physical media, there has been a recent push toward digitizing this process. Direct patient access to health data through application programming interfaces (APIs) is an important part of current United States policy initiatives, and Apple has created the product “Health Records on iPhone” to leverage APIs for this purpose.

**Objective:**

The objective of this study was to examine the characteristics of patients at our institution who connected their personal iPhone devices to our electronic health records (EHRs) system through “Health Records on iPhone”, as compared to patients at our institution who used our patient portal but did not connect a personal device to our system.

**Methods:**

We examined adult patients at our institution who had authorized an iPhone device to download their health data from the Partners HealthCare EHR via APIs through “Health Records on iPhone” from February 18, 2018 (the date this feature was enabled at our health system) until February 17, 2019. We compared these patients to adult patients who used our portal at least once during this period but did not authorize an iPhone device to download their data via APIs.

**Results:**

Variables associated with an increased likelihood of using “Health Records on iPhone” included male gender (adjusted OR 3.36; 95% CI 3.11-3.62; *P*<.001) and younger age, particularly below 50 years of age. With each decade of age over 50, people were less likely to be “Health Records on iPhone” product users. Asian patients were more likely to use the product than Caucasian patients (adjusted OR 1.32; 95% CI 1.16-1.51; *P*<.001), though there was no significant difference between African Americans and Caucasians (adjusted OR 1.15; 95% CI 0.94-1.41; *P*=.17). Patients who resided in higher ZIP code income quartiles were more likely to be users than those in the lowest quartile.

**Conclusions:**

Early results from the implementation of patient-facing APIs at a single institution suggest that there are opportunities for expanding these technologies to ensure all patients are aware of, and have access to, their health data on their personal devices. More work is needed on expanding these technologies to different patient populations.

## Introduction

Giving patients access to their own health data is widely felt to be beneficial for numerous reasons, including better patient engagement, enhanced care coordination, and improved patient safety [1-4]. In fact, defining an individual’s right to access their own health records represents an important component of the Health Insurance Portability and Accountability Act’s (HIPAA) Privacy Rule [5]. Personal health records (PHRs) are electronic applications, designed to be used by individuals, that allow for accessing, managing, and capturing health data about an individual. PHRs stand in contrast to electronic health records (EHRs), which are primarily used by clinicians and health care institutions, though EHRs may offer patients an online portal where they can access a subset of EHR information and enter additional information about their health [6]. PHRs have existed for decades, with many different implementations, from vendor products directly linked to EHRs to industry efforts led by companies like Google and Microsoft [1,6,7].

Notwithstanding the potential benefits of patients accessing their own health data electronically, PHR usage is often low, and as a result, many patients do not have electronic access to their health data [8-10]. Therefore, individual access to health records remains largely through paper and other physical media [11], even though physical records can be challenging to access. For example, in the United States, under HIPAA, hospitals can charge a fee for release of medical records which is often higher than recommended [12,13]. Regulatory noncompliance, procedural hurdles, and convenience issues with managing paper and physical media are additional barriers to patients accessing their health data [12,13].

Because of these concerns, the United States Centers for Medicare and Medicaid Services (CMS) Promoting Interoperability program (formerly Meaningful Use) has required various forms of electronic access to health data for patients for many years, with the most recent requirement that certified EHRs include functionality for patients to connect third-party applications via application programming interface (API) technology (a mechanism for applications to communicate directly with EHRs) [14]. Additionally, the 21st Century Cures Act (21CCA) requires that certified EHRs have published APIs available for patients [15] and the United States Department of Health and Human Services recently proposed a new rule that would implement these data sharing provisions from 21CCA and expand the number of required data elements to be shared [16].

With these regulations in mind, in 2018 Apple announced a new “Health Records on iPhone” feature that would enable patients to directly connect their iPhone through APIs to EHRs using a direct connection and would allow them to download, aggregate, and view their records (medications, allergies, results, etc). Patients could additionally choose to allow third-party apps to access these data [17]. The number of participating healthcare organizations has expanded substantially since the original release [18], and Apple recently expanded availability of this functionality to any US healthcare system with a compatible EHR [19]. Though this technology is limited to iPhone devices only, there are an estimated 193 million iPhone units in the United States [20], which presents a major opportunity for improving patient access to health data.

Despite the growing availability of third-party applications that connect to EHRs via APIs, little is known about the patients who have begun using them. Therefore, we examined the characteristics of patients at our institution who connected their personal Apple devices to our EHR through “Health Records on iPhone”, as compared to patients at our institution who used our patient portal but did not connect a personal device to our EHR.

## Methods

We identified adult patients at our institution who had authorized an iPhone device to download their health data via APIs with “Health Records on iPhone,” from the Partners HealthCare EHR, from February 18, 2018 (the date this feature was enabled at our health system) until February 17, 2019. For the purposes of this study, using the API was defined as authorizing the iPhone product “Health Records on iPhone” to download health data at least once during the study period. Authorizations were retrieved from an internal audit log database. Our healthcare system (Partners HealthCare) uses the Epic EHR software (Epic Systems Corporation, Verona, Wisconsin). During the study period, our portal was a custom-developed product that utilized native Epic MyChart functionality for many components, including the API functionality. Our control group consisted of adult patients who used our portal at least once during this period but did not authorize an iPhone device to download their data via APIs. The control group was not limited to patients with iPhone devices. Due to data use agreements, we were unable to report the total number of “Health Records on iPhone” users, so we instead took a random sample of the total from each population.

We calculated descriptive statistics and performed a multivariable logistic regression to compute odds ratios (ORs) with 95% CI for the odds that a patient would be in our case group (“Health Records on iPhone” users). Covariates included gender, age (split into ranges of 18-40, 41-50, 51-60, 61-70, and >80), race and ethnicity (using United States Census Bureau groupings), primary language, and the United States census median household income quartile of their primary ZIP code, which we obtained from the United States Census Bureau [21]. Patient characteristics data were obtained from an internal clinical reporting system. Data analysis was conducted using R statistical software version 3.5.1 (R Project for Statistical Computing, Vienna, Austria). The Partners HealthCare institutional review board approved this study.

## Results

We randomly sampled 3000 “Health Records on iPhone” users and compared them to 100,000 randomly sampled patient portal users who did not use the feature (Table 1).

**Table 1:** Association between patient characteristics and usage of “Health Records on iPhone”.

Characteristic		Non-“Health Records on iPhone” users (n=100,000), n (%)	“Health Records on iPhone” users (n=3000), n (%)	Adjusted OR^a^ (95% CI)	*P* value
**Gender**				3.36 (3.11-3.62)	<.001
	Female	62,813 (62.8)	1069 (35.6)	—^b^	—
	Male	37,187 (37.2)	1931 (64.4)	—	—
**Primary language**					
	English	95,251 (95.3)	2897 (96.6)	—	—
	Spanish	681 (0.68)	17 (0.57)	0.66 (0.40-1.11)	.12
	Other	1163 (1.16)	24 (0.80)	0.71 (0.47-1.07)	.11
	Not available	2905 (2.90)	62 (2.07)	0.83 (0.63-1.09)	.19
**Race**					
	Caucasian	83,215 (83.2)	2408 (80.3)	—	—
	Asian	5596 (5.60)	262 (8.73)	1.32 (1.16-1.51)	<.001
	African American	3196 (3.20)	109 (3.63)	1.15 (0.94-1.41)	.17
	Other	3390 (3.39)	124 (4.13)	0.91 (0.71-1.15)	.43
	Not available	4603 (4.60)	97 (3.23)	0.66 (0.52-0.83)	<.001
**Ethnicity**					
	Hispanic or Latino	3309 (3.31)	137 (4.57)	—	—
	Not Hispanic or Latino	88,092 (88.1)	2598 (86.6)	0.67 (0.53-0.85)	.001
	Not available	8599 (8.60)	265 (8.83)	0.77 (0.59-0.99)	.05
**Age range**					
	18-40	31,377 (31.4)	1233 (41.1)	—	—
	41-50	16,255 (16.3)	690 (23.0)	1.00 (0.90-1.10)	.94
	51-60	19,573 (19.6)	513 (17.1)	0.61 (0.54-0.67)	<.001
	61-70	18,844 (18.8)	363 (12.1)	0.43 (0.38-0.49)	<.001
	71-80	10,827 (10.8)	177 (5.90)	0.35 (0.39-0.41)	<.001
	>80	3124 (3.12)	24 (0.80)	0.16 (0.11-0.24)	<.001
**Median household income quartile, by ZIP code (US $)**					
	4836-41,406.50	24,844 (24.8)	669 (22.3)	—	—
	41,406.50-51,897	24,787 (24.8)	725 (24.2)	1.12 (1.01-1.25)	.05
	51,897-65,903.50	24,735 (24.7)	778 (25.9)	1.15 (1.03-1.28)	.02
	65,903.50-244,671	24,703 (24.7)	809 (27.0)	1.21 (1.09-1.35)	<.001
	Non-US, invalid, or missing	931 (0.93)	19 (0.63)	0.91 (0.57-1.45)	.71

^a^OR: odds ratio.

^b^Not applicable.

In a multivariable analysis, characteristics associated with an increased likelihood of using “Health Records on iPhone” included male gender (adjusted OR 3.36; 95% CI 3.11-3.62; *P*<.001) and younger age, particularly below 50 years of age. With each decade over 50 years of age, people were less likely to be “Health Records on iPhone” users. Additionally, Asian patients were more likely to use the app than Caucasians (adjusted OR 1.32; 95% CI 1.16-1.51; *P*<.001), though there was no significant difference between African Americans and Caucasians (adjusted OR 1.15; 95% CI 0.94-1.41; *P*=.17). Spanish as a primary language was not associated with “Health Records on iPhone” usage, as compared to English as a primary language (adjusted OR 0.66; 95% CI 0.40-1.11; *P*=.12). Hispanic ethnicity was more associated with “Health Records on iPhone” usage than non-Hispanic or non-Latino ethnicity (adjusted OR 0.67; 95% CI 0.53-0.85; *P*=.001). Finally, patients who resided in higher ZIP code income quartiles were more likely to be users than those in the lowest quartile, with comparisons to quartile 2 (adjusted OR 1.12; 95% CI 1.01-1.25; *P*=.05), to quartile 3 (adjusted OR 1.15; 95% CI 1.03-1.28; *P*=.02) and to quartile 4 (adjusted OR 1.21; 95% CI 1.09-1.35; *P*<.001). Full results are listed in Table 1. Unadjusted results did not substantially differ from the adjusted results and can be found in Multimedia Appendix 1.

## Discussion

In this single center study of the characteristics of patients who used “Health Records on iPhone”, patients that used the product differed in important ways from patients that also used our online patient portal but did not use the app. Initial users were more likely to be male and reside in a ZIP code with a higher median household income than patients who used our portal but did not connect their personal device to our EHR. Additionally, users of this technology were more likely to be younger than 50 compared to non-API portal users. Patients of Asian race used “Health Records on iPhone” more than patients of Caucasian race, but no other racial or ethnic differences were observed beyond non-Hispanic or non-Latino ethnicity using it less than Hispanic or Latino ethnicity.

In the United States, patients have the right to access their health information, as the HIPAA Privacy Rule requires that covered entities give individuals access to their data, upon request, with exceptions for some items like psychotherapy notes [22]. PHRs and patient portals have existed for decades in various forms, though there has been significant expansion of these technologies in the last decade driven by overall technological advancements and US federal regulatory requirements in the form of Meaningful Use (now called Promoting Interoperability). Prior work has shown that, overall, patient portal usage seems to be increasing [23,24] but remains low, with typically less than 50% of patients using online portals [9,10,25]. Additionally, prior work has shown that patient portal use may reflect or exacerbate a digital divide between sociodemographic patient groups. For example, Perzynski et al [25] showed that racial and ethnic minorities and patients of lower socioeconomic status were less likely to use a patient portal, along with those without broadband internet access in their neighborhood. Pho et al and Gerber et al [23,24] showed that in an oncology population, certain characteristics were more associated with portal usage, such as younger age, Caucasian race, and Spanish-speaking patients. Lockwood et al [9] also demonstrated important sociodemographic differences in portal usage in a pre and post-kidney transplant population. These, and our findings, exist within a broader literature describing a very real digital divide between patients who do not have ready access to the internet due to literacy, cost, or other barriers, and those that do have immediate access [26-31].

APIs provide an opportunity for deeper, more seamless integration with health data and EHRs than is possible through patient portals, and their availability is now required as part of a certified EHR. Through APIs an entire ecosystem of PHRs can form, with patients free to choose among multiple solutions depending on their needs. APIs are particularly enabling for mobile devices to retrieve health data, with these devices becoming increasingly common. For example, smartphone usage in the United States has dramatically increased over the past decade, from 33% in 2011 to 84% in 2019 [32]. More Americans now have a smartphone than a desktop or laptop computer [32]. “Health Records on iPhone” is one of the first major products to take advantage of these functionalities. In the United States, the CMS program Promoting Interoperability requires certified EHRs to include patient-facing API technology as of 2019. Because this technology is new, little is known about what types of patients are connecting their personal devices to the EHR to retrieve data. Initial reports suggest patients are receptive to these technologies [33], but we are not aware of any other work examining the characteristics of patients using APIs. Our results are an important first look at which patients are connecting their personal devices to the EHR system through APIs.

Our study has several limitations. First, we looked only at patient demographics. More work is needed to look at the clinical characteristics of these patients. Additionally, our study is a single site analysis and looked only at one product (“Health Records on iPhone”) on one type of smartphone operating system (Apple, iOS). “Health Records on iPhone” is by far the largest current implementation of these technologies. As these technologies expand, we expect more product usage across different types of personal devices, and it will be essential that these analyses are replicated for difference devices and populations. Third, we did not have access to the specific model of iPhone device used by the patients. Finally, we were unable to account for unmeasured clustering of patients using these technologies (eg, driven by specific provider groups that encouraged usage). However, we are not aware of any such behaviors at our institution.

In summary, we report here a first look at the characteristics of the initial cohort of patients who used “Health Records on iPhone” and show that these initial patients differ in important ways from patients who did not use this product but still used our portal. More work is needed to understand how to expand this technology to other members of the community and how policies can be modified to improve patient access to data broadly.

## Appendix

Multimedia Appendix 1 Unadjusted patient characteristics of health records on iPhone users compared to patient portal users who did not use health records on iPhone.

## Abbreviations

21CCA: 21st Century Cures Act
API: application programming interface
CMS: Centers for Medicare and Medicaid Services
EHR: electronic health record
HIPAA: Health Insurance Portability and Accountability Act
OR: odds ratio
PHR: personal health record
