# Supplementary material for: Comparing Characteristics of Patients Who Connect Their iPhones to an Electronic Health Records System Versus Patients Who Connect Without Personal Devices: Cohort Study
Source: J Med Internet Res. 2019 Aug 22;21(8):e14871. doi: 10.2196/14871 (PMC6727627; doi:10.2196/14871)
Supplement: Multimedia Appendix 1 [file jmir_v21i8e14871_app1.pdf]

Supplementary Table 1: Unadjusted Patient Characteristics of Health Records on iPhone Users Compared to Patient Portal Users Who Did Not Use Health Records on iPhone

	Non-API Users (n=100,000)	API Users (n=3,000)	OR [95% CI]	P value
Gender:				
Female	62813 (62.8%)	1069 (35.6%)	Ref.	Ref.
Male	37187 (37.2%)	1931 (64.4%)	3.05 [2.83;3.29]	<0.001
Primary Language:				
English	95251 (95.3%)	2897 (96.6%)	Ref.	Ref.
Spanish	681 (0.68%)	17 (0.57%)	0.83 [0.49;1.30]	0.432
Other	1163 (1.16%)	24 (0.80%)	0.68 [0.44;1.00]	0.050
Not Available	2905 (2.90%)	62 (2.07%)	0.70 [0.54;0.90]	0.004
Race:				
White	83215 (83.2%)	2408 (80.3%)	Ref.	Ref.
Asian	5596 (5.60%)	262 (8.73%)	1.62 [1.42;1.84]	<0.001
Black or African American	3196 (3.20%)	109 (3.63%)	1.18 [0.97;1.43]	0.104
Other	3390 (3.39%)	124 (4.13%)	1.27 [1.05;1.51]	0.015
Not Available	4603 (4.60%)	97 (3.23%)	0.73 [0.59;0.89]	0.002
Ethnicity:				
Hispanic or Latino	3309 (3.31%)	137 (4.57%)	Ref.	Ref.
Not Hispanic or Latino	88092 (88.1%)	2598 (86.6%)	0.71 [0.60;0.85]	<0.001
Not Available	8599 (8.60%)	265 (8.83%)	0.74 [0.60;0.92]	0.007
Age:				
18-40	31377 (31.4%)	1233 (41.1%)	Ref.	Ref.
41-50	16255 (16.3%)	690 (23.0%)	1.08 [0.98;1.19]	0.113
51-60	19573 (19.6%)	513 (17.1%)	0.67 [0.60;0.74]	<0.001
61-70	18844 (18.8%)	363 (12.1%)	0.49 [0.44;0.55]	<0.001
71-80	10827 (10.8%)	177 (5.90%)	0.42 [0.35;0.49]	<0.001
> 80	3124 (3.12%)	24 (0.80%)	0.20 [0.13;0.29]	<0.001
Median Household Income Quartile, By Zip Code (USD):				
$4,836 - $41,406.50	24844 (24.8%)	669 (22.3%)	Ref.	Ref.
$41,406.50 - $51,897	24787 (24.8%)	725 (24.2%)	1.09 [0.98;1.21]	0.128
$51,897 - $65,903.50	24735 (24.7%)	778 (25.9%)	1.17 [1.05;1.30]	0.004
$65,903.50 - $244,671	24703 (24.7%)	809 (27.0%)	1.22 [1.10;1.35]	<0.001

Non-US, Invalid, or Missing	931 (0.93%)	19 (0.63%)	0.76 [0.47;1.18]	0.233
